# Tetra­kis(μ_2_-2,2-dimethyl­propanoato-κ^2^ *O*,*O*′)bis­[(pyridine-κ*N*)copper(II)]: a monoclinic polymorph

**DOI:** 10.1107/S1600536810015060

**Published:** 2010-04-30

**Authors:** Lailatun Nazirah Ozair, Norbani Abdullah, Hamid Khaledi, Edward R. T. Tiekink

**Affiliations:** a Department of Chemistry, University of Malaya, 50603 Kuala Lumpur, Malaysia

## Abstract

The structure of the dinuclear title complex, [Cu_2_(C_5_H_9_O_2_)_4_(C_5_H_5_N)_2_], represents a monoclinic polymorph of the previously reported triclinic form [Blewett *et al.* (2006 ▶). *Acta Cryst*. E**62**, m420–m422]. Each carboxyl­ate group is bidentate bridging and the distorted octa­hedral geometry about each Cu^II^ atom is completed by a pyridine N atom and the other Cu atom [Cu⋯Cu = 2.6139 (7) Å]. In the crystal, mol­ecules are connected into supra­molecular chains *via* π–π inter­actions formed by the pyridine rings [centroid–centroid distance = 3.552 (3) Å] and these are connected into a two-dimensional array in the *ac* plane by C—H⋯π contacts. One of the *tert*-butyl groups is disordered over two orientations in a 0.734 (6):0.266 (6) ratio.

## Related literature

For the structure of the triclinic polymorph of the title compound, see: Blewett *et al.* (2006 ▶). For background to copper(II) carboxyl­ates, see: Attard & Cullum (1990 ▶); Kato *et al.* (1964 ▶); Melnik *et al.* (1984 ▶); Kawata *et al.* (1992 ▶).
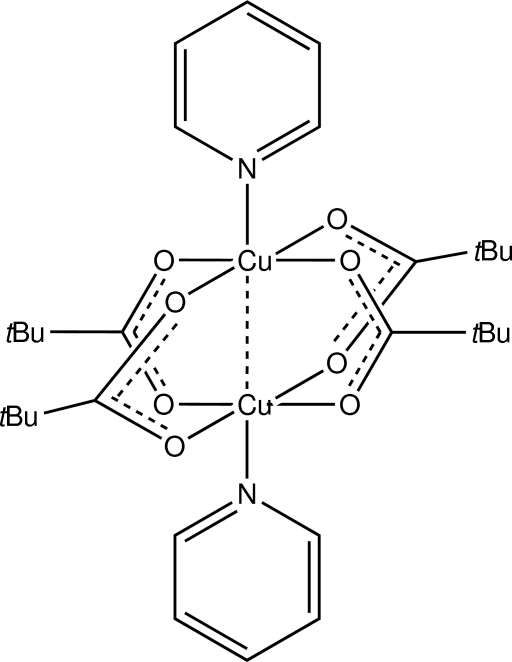


## Experimental

### 

#### Crystal data

[Cu_2_(C_5_H_9_O_2_)_4_(C_5_H_5_N)_2_]
*M* *_r_* = 689.80
Monoclinic, 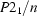

*a* = 9.4758 (6) Å
*b* = 20.0192 (12) Å
*c* = 18.6136 (10) Å
β = 104.515 (3)°
*V* = 3418.3 (4) Å^3^
*Z* = 4
Mo *K*α radiation
μ = 1.29 mm^−1^
*T* = 100 K
0.32 × 0.26 × 0.16 mm

#### Data collection

Bruker SMART APEX CCD diffractometer
Absorption correction: multi-scan (*SADABS*; Sheldrick, 1996 ▶) *T*_min_ = 0.682, *T*_max_ = 0.820
28775 measured reflections
7077 independent reflections
5583 reflections with *I* > 2σ(*I*)
*R*_int_ = 0.060

#### Refinement

*R*[*F* ^2^ > 2σ(*F* ^2^)] = 0.058
*wR*(*F* ^2^) = 0.156
*S* = 1.13
7077 reflections
404 parameters
12 restraints
H-atom parameters constrained
Δρ_max_ = 1.26 e Å^−3^
Δρ_min_ = −0.75 e Å^−3^

### 

Data collection: *APEX2* (Bruker, 2008 ▶); cell refinement: *SAINT* (Bruker, 2008 ▶); data reduction: *SAINT*; program(s) used to solve structure: *SHELXS97* (Sheldrick, 2008 ▶); program(s) used to refine structure: *SHELXL97* (Sheldrick, 2008 ▶); molecular graphics: *ORTEP-3* (Farrugia, 1997 ▶) and *DIAMOND* (Brandenburg, 2006 ▶); software used to prepare material for publication: *publCIF* (Westrip, 2010 ▶).

## Supplementary Material

Crystal structure: contains datablocks global, I. DOI: 10.1107/S1600536810015060/hb5416sup1.cif

Structure factors: contains datablocks I. DOI: 10.1107/S1600536810015060/hb5416Isup2.hkl

Additional supplementary materials:  crystallographic information; 3D view; checkCIF report

## Figures and Tables

**Table 1:** Selected bond lengths (Å)

Cu1—O7	1.950 (3)
Cu1—O1	1.956 (3)
Cu1—O3	1.976 (3)
Cu1—O5	1.987 (3)
Cu1—N1	2.157 (3)
Cu2—O6	1.962 (3)
Cu2—O4	1.968 (3)
Cu2—O8	1.976 (3)
Cu2—O2	1.978 (3)
Cu2—N2	2.157 (3)

**Table 2:** Hydrogen-bond geometry (Å, °) *Cg*1 and *Cg*2 are the centroids of the N1,C21–C25 and N2,C26–C30 rings, respectively.

*D*—H⋯*A*	*D*—H	H⋯*A*	*D*⋯*A*	*D*—H⋯*A*
C3—H3c⋯*Cg*1^i^	0.98	2.90	3.609 (7)	130
C19b—H19f⋯*Cg*2^ii^	0.98	2.64	3.554 (19)	154

Symmetry codes: (i) 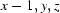; (ii) 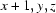.

**Fig. 1.**
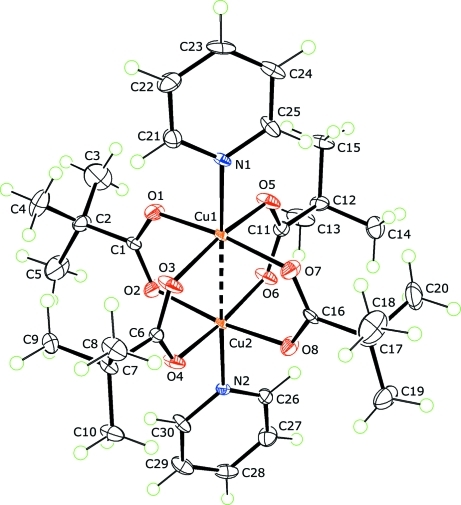
The molecular structure of (I) showing displacement ellipsoids at the 50% probability level. Only the major component of the disordered tert-butyl group is shown for reasons of clarity.

**Fig. 2.**
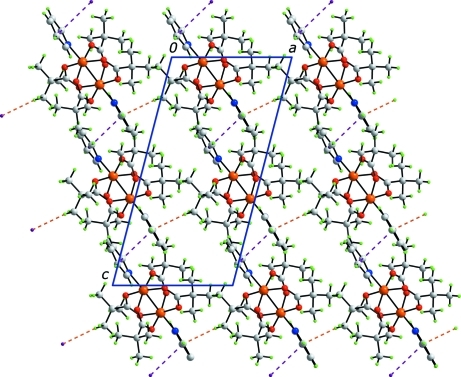
The 2-D array in the ac plane in (I) mediated by π–π and C–H···π interactions, shown as purple and orange dashed lines, respectively. Colour code: Cu, orange; O, red; N, blue; C, grey; and H, green.

**Fig. 3.**
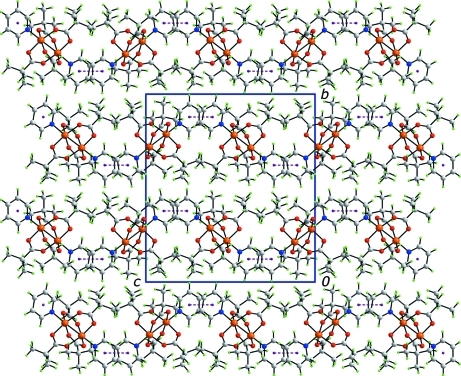
Packing of layers in (I) along the b axis. Colour code: Cu, orange; O, red; N, blue; C, grey; and H, green.

## supplementary crystallographic information

### Comment

Research on copper(II) carboxylates focuses upon their metallomesogenic properties (Attard & Cullum, 1990) and interesting magneto-structural relationship (Kato *et al.*, 1964; Melnik *et al.*, 1984; Kawata *et al.*, 1992). However, the practical use of these complexes is hindered by their high melting points (greater than 523 K) and which are accompanied by thermal decomposition. Our research interest is to develop low-temperature copper(II) carboxylates as functional materials for use in the fields of catalysis, photonics, spintronics, and electronics. To realise this, we adopted the concepts of symmetry reduction by mixed ligands and the use of highly-branched alkylcarboxylates. This contribution reports the crystal structure of one of the starting materials to be used in the synthesis of such complexes, *i.e*. the title compound, (I).

The dinuclear structure of (I), Fig. 1, features two Cu atoms, separated by 2.6139 (7) Å, connected by four bidentate bridging carboxylate ligands. The final position in the disordered octahedral *trans*-CuNO_4_ donor set is occupied by a pyridine-N atom in each case. The structure resembles closely that described for the triclinic polymorph but with the latter being disposed about a centre of inversion (Blewett *et al.*, 2006). The primary differences between the molecules is found in the relative disposition of the pyridine groups. In (I), the dihedral angles formed between the least-squares planes through the four O atoms and pyridine ring = 81.5 (1) ° for Cu1 and 88.6 (1) ° for Cu2, which compares to 89.38 (8) ° found in the triclinic structure. These differences are reflected in the dihedral angle of 12.93 (15) ° formed between the pyridine rings in (I) compared to 0 ° (from symmetry) in the triclinic polymorph. These differences not withstanding, the Cu–O bond distances are experimentally equivalent in the two forms but it is noted these cover are broader range in (I), *i.e*. 1.950 (3) to 1.987 (3) Å, compared with 1.963 (2) to 1.977 (2) Å; the Cu–N distances are indistinguishable. The Cu···Cu distance in (I), 2.6139 (7) Å, is shorter than 2.6229 (9) Å in the triclinic form.

A common feature of the crystal packing of both forms is the presence of significant π–π interactions between the pyridine rings. In (I), these [ring centroid(N1,C21—C25)···ring centroid(N2,C26—C30)^i^ = 3.552 (3) Å, angle between planes = 9.2 (2) °, for *i*: 1/2+*x*, 1/2-*y*, 1/2+*z*] lead to supramolecular chains which are connected into a 2-D array in the *ac* plane by C–H···π contacts involving methyl-H atoms (one being derived from a disordered *tert*-butyl residue), Fig. 2 & Table 1. The layers are stacked along the *b* direction as illustrated in Fig. 3.

### Experimental

An aqueous solution (50 ml) of sodium carbonate (5.2 g, 0.049 mol) was added to an aqueous solution (50 ml) of 2,2-dimethylpropionic acid (10 g, 0.098 mol) and the mixture was stirred at 323 K. After 30 min, a solution of CuCl_2_.2H_2_O (8.33 g, 0.049 mol) dissolved in a minimum amount of water was added followed by addition of several drops of pyridine. The mixture was stirred for another 30 min. and then set aside at room temperature for a week whereupon green blocks of (I) were obtained. Both DSC and TGA data indicate that the material did not melt, but decomposed at 408 K. CHN analyses (%), Found: C, 52.17; H, 6.75; N, 4.16. Calc'd: C, 52.23; H, 6.72; N, 4.05.

### Refinement

Carbon-bound H-atoms were placed in calculated positions (C—H 0.95 to 0.98 Å) and were included in the refinement in the riding model approximation, with *U*_iso_(H) set to 1.2 to 1.5*U*_equiv_(C). One of the *tert*-butyl groups was found to be disordered with two positions being resolved for each of the methyl groups. From anisotropic refinement, the major component of the disorder had a site occupancy factor = 0.734 (6). The C–C bond distances for the disordered group were refined with the distance restraint 1.52±0.01 Å, and the anisotropic displacement parameters for pairs of disordered atoms were constrained to be equivalent with the EADP command in SHELXL-97 (Sheldrick, 2008). The maximum and minimum residual electron density peaks of 1.26 and 0.75 e Å^-3^, respectively, were located 1.43 Å and 0.24 Å from the H29 and C19b atoms, respectively.

### Crystal data

**Table:**

[Cu_2_(C_5_H_9_O_2_)_4_(C_5_H_5_N)_2_]	*F*(000) = 1448
*M**_r_* = 689.80	*D*_x_ = 1.340 Mg m^−^^3^
Monoclinic, *P*2_1_/*n*	Mo *K*α radiation, λ = 0.71073 Å
Hall symbol: -P 2yn	Cell parameters from 6465 reflections
*a* = 9.4758 (6) Å	θ = 2.2–28.4°
*b* = 20.0192 (12) Å	µ = 1.29 mm^−^^1^
*c* = 18.6136 (10) Å	*T* = 100 K
β = 104.515 (3)°	Block, green
*V* = 3418.3 (4) Å^3^	0.32 × 0.26 × 0.16 mm
*Z* = 4	

### Data collection

**Table:**

Bruker SMART APEX CCD diffractometer	7077 independent reflections
Radiation source: fine-focus sealed tube	5583 reflections with *I* > 2σ(*I*)
graphite	*R*_int_ = 0.060
ω scans	θ_max_ = 26.5°, θ_min_ = 1.5°
Absorption correction: multi-scan (*SADABS*; Sheldrick, 1996)	*h* = −11→11
*T*_min_ = 0.682, *T*_max_ = 0.820	*k* = −25→25
28775 measured reflections	*l* = −23→23

### Refinement

**Table:**

Refinement on *F*^2^	Primary atom site location: structure-invariant direct methods
Least-squares matrix: full	Secondary atom site location: difference Fourier map
*R*[*F*^2^ > 2σ(*F*^2^)] = 0.058	Hydrogen site location: inferred from neighbouring sites
*wR*(*F*^2^) = 0.156	H-atom parameters constrained
*S* = 1.13	*w* = 1/[σ^2^(*F*_o_^2^) + (0.0532*P*)^2^ + 12.7516*P*] where *P* = (*F*_o_^2^ + 2*F*_c_^2^)/3
7077 reflections	(Δ/σ)_max_ = 0.001
404 parameters	Δρ_max_ = 1.26 e Å^−^^3^
12 restraints	Δρ_min_ = −0.75 e Å^−^^3^

### Special details

**Table:**

Geometry. All s.u.'s (except the s.u. in the dihedral angle between two l.s. planes) are estimated using the full covariance matrix. The cell s.u.'s are taken into account individually in the estimation of s.u.'s in distances, angles and torsion angles; correlations between s.u.'s in cell parameters are only used when they are defined by crystal symmetry. An approximate (isotropic) treatment of cell s.u.'s is used for estimating s.u.'s involving l.s. planes.
Refinement. Refinement of *F*^2^ against ALL reflections. The weighted *R*-factor wR and goodness of fit *S* are based on *F*^2^, conventional *R*-factors *R* are based on *F*, with *F* set to zero for negative *F*^2^. The threshold expression of *F*^2^ > 2σ(*F*^2^) is used only for calculating *R*-factors(gt) etc. and is not relevant to the choice of reflections for refinement. *R*-factors based on *F*^2^ are statistically about twice as large as those based on *F*, and *R*- factors based on ALL data will be even larger.

### Fractional atomic coordinates and isotropic or equivalent isotropic displacement parameters (Å^2^)

**Table:**

	*x*	*y*	*z*	*U*_iso_*/*U*_eq_	Occ. (<1)
Cu1	0.45858 (5)	0.21308 (2)	0.12035 (3)	0.01076 (14)	
Cu2	0.26828 (5)	0.28113 (2)	0.02145 (3)	0.01059 (14)	
O1	0.2929 (3)	0.15985 (17)	0.13229 (18)	0.0249 (8)	
O2	0.1310 (3)	0.21490 (17)	0.04446 (18)	0.0234 (7)	
O3	0.4621 (4)	0.15777 (16)	0.03288 (17)	0.0246 (8)	
O4	0.3063 (4)	0.21771 (15)	−0.05224 (16)	0.0206 (7)	
O5	0.4194 (4)	0.27884 (16)	0.19295 (16)	0.0227 (7)	
O6	0.2568 (3)	0.33601 (16)	0.10715 (16)	0.0219 (7)	
O7	0.5986 (3)	0.27532 (16)	0.09663 (18)	0.0233 (7)	
O8	0.4386 (3)	0.33408 (16)	0.01196 (18)	0.0217 (7)	
N1	0.6203 (4)	0.15694 (17)	0.19951 (18)	0.0127 (7)	
N2	0.1082 (4)	0.34034 (17)	−0.05504 (18)	0.0114 (7)	
C1	0.1662 (5)	0.1693 (2)	0.0920 (2)	0.0162 (9)	
C2	0.0475 (5)	0.1208 (2)	0.1027 (3)	0.0229 (10)	
C3	0.0191 (7)	0.1362 (4)	0.1771 (3)	0.0458 (16)	
H3A	−0.0212	0.1813	0.1763	0.069*	
H3B	0.1106	0.1334	0.2157	0.069*	
H3C	−0.0507	0.1038	0.1876	0.069*	
C4	0.1053 (7)	0.0488 (3)	0.1029 (4)	0.0405 (14)	
H4A	0.1948	0.0440	0.1426	0.061*	
H4B	0.1262	0.0393	0.0549	0.061*	
H4C	0.0317	0.0175	0.1112	0.061*	
C5	−0.0901 (6)	0.1281 (4)	0.0406 (4)	0.0524 (19)	
H5A	−0.1589	0.0925	0.0446	0.079*	
H5B	−0.0656	0.1246	−0.0074	0.079*	
H5C	−0.1347	0.1716	0.0444	0.079*	
C6	0.3892 (4)	0.1687 (2)	−0.0318 (2)	0.0134 (8)	
C7	0.3937 (5)	0.1150 (2)	−0.0905 (2)	0.0151 (9)	
C8	0.5456 (5)	0.0838 (3)	−0.0754 (3)	0.0260 (11)	
H8A	0.5710	0.0643	−0.0255	0.039*	
H8B	0.6170	0.1183	−0.0789	0.039*	
H8C	0.5462	0.0488	−0.1121	0.039*	
C9	0.2810 (5)	0.0621 (2)	−0.0821 (3)	0.0232 (10)	
H9A	0.3111	0.0419	−0.0326	0.035*	
H9B	0.2749	0.0273	−0.1199	0.035*	
H9C	0.1854	0.0832	−0.0884	0.035*	
C10	0.3515 (5)	0.1443 (2)	−0.1687 (2)	0.0230 (10)	
H10A	0.3551	0.1091	−0.2049	0.035*	
H10B	0.4198	0.1800	−0.1728	0.035*	
H10C	0.2525	0.1625	−0.1787	0.035*	
C11	0.3319 (5)	0.3263 (2)	0.1724 (2)	0.0146 (8)	
C12	0.3165 (5)	0.3794 (2)	0.2300 (2)	0.0188 (9)	
C13	0.1568 (6)	0.3994 (3)	0.2176 (3)	0.0307 (12)	
H13A	0.1483	0.4348	0.2526	0.046*	
H13B	0.0997	0.3605	0.2256	0.046*	
H13C	0.1200	0.4156	0.1667	0.046*	
C14	0.4075 (6)	0.4394 (3)	0.2171 (3)	0.0332 (12)	
H14A	0.3725	0.4548	0.1657	0.050*	
H14B	0.5100	0.4262	0.2263	0.050*	
H14C	0.3980	0.4756	0.2510	0.050*	
C15	0.3755 (5)	0.3530 (3)	0.3093 (2)	0.0244 (10)	
H15A	0.3713	0.3886	0.3449	0.037*	
H15B	0.4767	0.3385	0.3160	0.037*	
H15C	0.3161	0.3151	0.3177	0.037*	
C16	0.5650 (5)	0.3216 (2)	0.0502 (2)	0.0140 (8)	
C17A	0.6897 (5)	0.3671 (2)	0.0410 (2)	0.0239 (10)	0.734 (6)
C18A	0.8277 (7)	0.3287 (4)	0.0437 (5)	0.0402 (19)	0.734 (6)
H18A	0.8152	0.3020	−0.0016	0.060*	0.734 (6)
H18B	0.8489	0.2990	0.0870	0.060*	0.734 (6)
H18C	0.9087	0.3600	0.0475	0.060*	0.734 (6)
C19A	0.6466 (8)	0.4147 (4)	−0.0237 (4)	0.0373 (19)	0.734 (6)
H19A	0.7232	0.4483	−0.0203	0.056*	0.734 (6)
H19B	0.5550	0.4369	−0.0226	0.056*	0.734 (6)
H19C	0.6336	0.3898	−0.0702	0.056*	0.734 (6)
C20A	0.7221 (9)	0.4117 (4)	0.1122 (4)	0.0397 (19)	0.734 (6)
H20A	0.7620	0.3840	0.1559	0.060*	0.734 (6)
H20B	0.6317	0.4329	0.1169	0.060*	0.734 (6)
H20C	0.7930	0.4463	0.1082	0.060*	0.734 (6)
C17B	0.6897 (5)	0.3671 (2)	0.0410 (2)	0.0239 (10)	0.266 (6)
C18B	0.8322 (14)	0.3532 (12)	0.0968 (11)	0.0402 (19)	0.266 (6)
H18D	0.9104	0.3785	0.0835	0.060*	0.266 (6)
H18E	0.8540	0.3053	0.0969	0.060*	0.266 (6)
H18F	0.8247	0.3666	0.1464	0.060*	0.266 (6)
C19B	0.711 (2)	0.3343 (10)	−0.0311 (8)	0.0373 (19)	0.266 (6)
H19D	0.6414	0.3536	−0.0741	0.056*	0.266 (6)
H19E	0.6943	0.2860	−0.0295	0.056*	0.266 (6)
H19F	0.8106	0.3425	−0.0353	0.056*	0.266 (6)
C20B	0.636 (2)	0.4363 (6)	0.0178 (13)	0.0397 (19)	0.266 (6)
H20D	0.5969	0.4566	0.0568	0.060*	0.266 (6)
H20E	0.5582	0.4339	−0.0283	0.060*	0.266 (6)
H20F	0.7162	0.4635	0.0097	0.060*	0.266 (6)
C21	0.6186 (5)	0.0903 (2)	0.2011 (3)	0.0212 (10)	
H21	0.5543	0.0671	0.1615	0.025*	
C22	0.7063 (6)	0.0535 (2)	0.2577 (3)	0.0266 (11)	
H22	0.7017	0.0061	0.2572	0.032*	
C23	0.8012 (5)	0.0870 (3)	0.3153 (2)	0.0247 (11)	
H23	0.8628	0.0629	0.3549	0.030*	
C24	0.8046 (5)	0.1551 (3)	0.3142 (2)	0.0234 (10)	
H24	0.8681	0.1793	0.3531	0.028*	
C25	0.7131 (5)	0.1884 (2)	0.2548 (2)	0.0215 (10)	
H25	0.7170	0.2358	0.2536	0.026*	
C26	0.0947 (5)	0.4055 (2)	−0.0447 (2)	0.0173 (9)	
H26	0.1561	0.4256	−0.0020	0.021*	
C27	−0.0046 (5)	0.4454 (2)	−0.0933 (3)	0.0246 (10)	
H27	−0.0134	0.4915	−0.0833	0.030*	
C28	−0.0911 (5)	0.4162 (3)	−0.1571 (3)	0.0259 (11)	
H28	−0.1586	0.4425	−0.1922	0.031*	
C29	−0.0778 (5)	0.3487 (3)	−0.1689 (3)	0.0242 (10)	
H29	−0.1361	0.3276	−0.2119	0.029*	
C30	0.0230 (5)	0.3123 (2)	−0.1163 (2)	0.0182 (9)	
H30	0.0320	0.2657	−0.1239	0.022*	

### Atomic displacement parameters (Å^2^)

**Table:**

	*U*^11^	*U*^22^	*U*^33^	*U*^12^	*U*^13^	*U*^23^
Cu1	0.0098 (2)	0.0124 (3)	0.0083 (2)	−0.00001 (19)	−0.00105 (18)	0.00013 (18)
Cu2	0.0095 (2)	0.0119 (3)	0.0086 (2)	−0.00012 (19)	−0.00098 (18)	0.00001 (18)
O1	0.0145 (16)	0.0320 (19)	0.0222 (17)	−0.0075 (14)	−0.0069 (13)	0.0127 (14)
O2	0.0133 (15)	0.0284 (18)	0.0257 (17)	−0.0046 (13)	−0.0005 (13)	0.0113 (14)
O3	0.0311 (19)	0.0243 (18)	0.0120 (15)	0.0147 (14)	−0.0063 (13)	−0.0042 (13)
O4	0.0292 (18)	0.0195 (16)	0.0103 (14)	0.0108 (14)	−0.0002 (13)	−0.0005 (12)
O5	0.0270 (18)	0.0248 (18)	0.0126 (15)	0.0133 (14)	−0.0021 (13)	−0.0028 (13)
O6	0.0237 (17)	0.0254 (17)	0.0123 (15)	0.0105 (14)	−0.0032 (13)	−0.0040 (13)
O7	0.0134 (15)	0.0272 (18)	0.0266 (17)	−0.0039 (13)	−0.0001 (13)	0.0112 (14)
O8	0.0130 (15)	0.0225 (17)	0.0266 (17)	−0.0031 (13)	−0.0010 (13)	0.0084 (13)
N1	0.0112 (17)	0.0171 (18)	0.0079 (16)	0.0042 (14)	−0.0012 (13)	−0.0012 (13)
N2	0.0096 (16)	0.0158 (17)	0.0089 (16)	−0.0004 (13)	0.0022 (13)	0.0010 (13)
C1	0.019 (2)	0.019 (2)	0.0090 (19)	−0.0055 (17)	0.0001 (16)	−0.0002 (16)
C2	0.017 (2)	0.029 (3)	0.021 (2)	−0.0090 (19)	0.0013 (18)	0.0088 (19)
C3	0.036 (3)	0.072 (5)	0.037 (3)	−0.017 (3)	0.023 (3)	−0.005 (3)
C4	0.046 (4)	0.026 (3)	0.052 (4)	−0.013 (3)	0.017 (3)	0.006 (3)
C5	0.027 (3)	0.060 (4)	0.055 (4)	−0.026 (3)	−0.018 (3)	0.026 (3)
C6	0.013 (2)	0.014 (2)	0.0116 (19)	−0.0021 (16)	0.0015 (16)	0.0010 (16)
C7	0.014 (2)	0.019 (2)	0.0113 (19)	0.0026 (17)	0.0004 (16)	−0.0039 (16)
C8	0.025 (3)	0.028 (3)	0.024 (2)	0.010 (2)	0.003 (2)	−0.005 (2)
C9	0.031 (3)	0.018 (2)	0.020 (2)	−0.006 (2)	0.006 (2)	−0.0052 (18)
C10	0.029 (3)	0.026 (3)	0.012 (2)	0.008 (2)	0.0003 (19)	−0.0005 (18)
C11	0.015 (2)	0.016 (2)	0.014 (2)	0.0002 (17)	0.0057 (17)	−0.0002 (16)
C12	0.026 (2)	0.014 (2)	0.015 (2)	0.0071 (18)	0.0036 (18)	0.0018 (17)
C13	0.028 (3)	0.044 (3)	0.019 (2)	0.020 (2)	0.003 (2)	0.000 (2)
C14	0.047 (3)	0.022 (3)	0.029 (3)	−0.008 (2)	0.008 (2)	−0.007 (2)
C15	0.031 (3)	0.030 (3)	0.010 (2)	0.012 (2)	0.0025 (19)	−0.0015 (18)
C16	0.014 (2)	0.020 (2)	0.0075 (18)	−0.0022 (17)	0.0009 (16)	−0.0057 (16)
C17A	0.015 (2)	0.035 (3)	0.022 (2)	−0.002 (2)	0.0027 (19)	0.007 (2)
C18A	0.021 (3)	0.044 (5)	0.062 (5)	0.001 (3)	0.022 (4)	0.008 (4)
C19A	0.019 (3)	0.048 (4)	0.041 (4)	−0.011 (3)	0.001 (3)	0.021 (4)
C20A	0.039 (4)	0.047 (5)	0.035 (4)	−0.028 (4)	0.012 (3)	−0.013 (3)
C17B	0.015 (2)	0.035 (3)	0.022 (2)	−0.002 (2)	0.0027 (19)	0.007 (2)
C18B	0.021 (3)	0.044 (5)	0.062 (5)	0.001 (3)	0.022 (4)	0.008 (4)
C19B	0.019 (3)	0.048 (4)	0.041 (4)	−0.011 (3)	0.001 (3)	0.021 (4)
C20B	0.039 (4)	0.047 (5)	0.035 (4)	−0.028 (4)	0.012 (3)	−0.013 (3)
C21	0.024 (2)	0.017 (2)	0.020 (2)	0.0008 (19)	0.0002 (18)	−0.0012 (18)
C22	0.033 (3)	0.018 (2)	0.027 (3)	0.008 (2)	0.003 (2)	0.0081 (19)
C23	0.017 (2)	0.039 (3)	0.015 (2)	0.012 (2)	−0.0027 (18)	0.011 (2)
C24	0.017 (2)	0.040 (3)	0.010 (2)	0.004 (2)	−0.0015 (17)	−0.0037 (19)
C25	0.019 (2)	0.023 (2)	0.018 (2)	0.0034 (19)	−0.0050 (18)	−0.0082 (18)
C26	0.016 (2)	0.017 (2)	0.016 (2)	−0.0021 (17)	−0.0009 (17)	0.0010 (17)
C27	0.023 (2)	0.019 (2)	0.030 (3)	0.0036 (19)	0.003 (2)	0.004 (2)
C28	0.023 (2)	0.036 (3)	0.016 (2)	0.008 (2)	0.0007 (19)	0.009 (2)
C29	0.016 (2)	0.036 (3)	0.017 (2)	0.006 (2)	−0.0017 (18)	−0.004 (2)
C30	0.013 (2)	0.024 (2)	0.015 (2)	0.0032 (18)	−0.0026 (17)	−0.0068 (17)

### Geometric parameters (Å, °)

**Table:**

Cu1—O7	1.950 (3)	C13—H13A	0.9800
Cu1—O1	1.956 (3)	C13—H13B	0.9800
Cu1—O3	1.976 (3)	C13—H13C	0.9800
Cu1—O5	1.987 (3)	C14—H14A	0.9800
Cu1—N1	2.157 (3)	C14—H14B	0.9800
Cu1—Cu2	2.6139 (7)	C14—H14C	0.9800
Cu2—O6	1.962 (3)	C15—H15A	0.9800
Cu2—O4	1.968 (3)	C15—H15B	0.9800
Cu2—O8	1.976 (3)	C15—H15C	0.9800
Cu2—O2	1.978 (3)	C16—C17B	1.535 (6)
Cu2—N2	2.157 (3)	C16—C17A	1.535 (6)
O1—C1	1.261 (5)	C17A—C18A	1.506 (7)
O2—C1	1.257 (5)	C17A—C19A	1.510 (6)
O3—C6	1.247 (5)	C17A—C20A	1.563 (7)
O4—C6	1.255 (5)	C18A—H18A	0.9800
O5—C11	1.256 (5)	C18A—H18B	0.9800
O6—C11	1.260 (5)	C18A—H18C	0.9800
O7—C16	1.253 (5)	C19A—H19A	0.9800
O8—C16	1.255 (5)	C19A—H19B	0.9800
N1—C25	1.332 (5)	C19A—H19C	0.9800
N1—C21	1.335 (6)	C20A—H20A	0.9800
N2—C26	1.329 (5)	C20A—H20B	0.9800
N2—C30	1.343 (5)	C20A—H20C	0.9800
C1—C2	1.535 (6)	C17B—C20B	1.503 (10)
C2—C3	1.506 (7)	C17B—C18B	1.508 (9)
C2—C5	1.518 (7)	C17B—C19B	1.553 (9)
C2—C4	1.541 (8)	C18B—H18D	0.9800
C3—H3A	0.9800	C18B—H18E	0.9800
C3—H3B	0.9800	C18B—H18F	0.9800
C3—H3C	0.9800	C19B—H19D	0.9800
C4—H4A	0.9800	C19B—H19E	0.9800
C4—H4B	0.9800	C19B—H19F	0.9800
C4—H4C	0.9800	C20B—H20D	0.9800
C5—H5A	0.9800	C20B—H20E	0.9800
C5—H5B	0.9800	C20B—H20F	0.9800
C5—H5C	0.9800	C21—C22	1.379 (6)
C6—C7	1.541 (6)	C21—H21	0.9500
C7—C10	1.527 (6)	C22—C23	1.388 (7)
C7—C8	1.530 (6)	C22—H22	0.9500
C7—C9	1.540 (6)	C23—C24	1.364 (7)
C8—H8A	0.9800	C23—H23	0.9500
C8—H8B	0.9800	C24—C25	1.392 (6)
C8—H8C	0.9800	C24—H24	0.9500
C9—H9A	0.9800	C25—H25	0.9500
C9—H9B	0.9800	C26—C27	1.383 (6)
C9—H9C	0.9800	C26—H26	0.9500
C10—H10A	0.9800	C27—C28	1.391 (7)
C10—H10B	0.9800	C27—H27	0.9500
C10—H10C	0.9800	C28—C29	1.380 (7)
C11—C12	1.542 (6)	C28—H28	0.9500
C12—C13	1.526 (6)	C29—C30	1.391 (6)
C12—C14	1.532 (7)	C29—H29	0.9500
C12—C15	1.535 (6)	C30—H30	0.9500
			
O7—Cu1—O1	170.12 (13)	C7—C10—H10C	109.5
O7—Cu1—O3	91.07 (15)	H10A—C10—H10C	109.5
O1—Cu1—O3	88.17 (15)	H10B—C10—H10C	109.5
O7—Cu1—O5	89.11 (15)	O5—C11—O6	125.5 (4)
O1—Cu1—O5	89.51 (15)	O5—C11—C12	118.7 (4)
O3—Cu1—O5	167.45 (12)	O6—C11—C12	115.8 (4)
O7—Cu1—N1	94.62 (13)	C13—C12—C14	110.2 (4)
O1—Cu1—N1	95.25 (13)	C13—C12—C15	110.1 (4)
O3—Cu1—N1	96.60 (13)	C14—C12—C15	109.7 (4)
O5—Cu1—N1	95.89 (12)	C13—C12—C11	109.8 (4)
O7—Cu1—Cu2	84.03 (9)	C14—C12—C11	106.1 (4)
O1—Cu1—Cu2	86.11 (9)	C15—C12—C11	110.9 (3)
O3—Cu1—Cu2	82.12 (9)	C12—C13—H13A	109.5
O5—Cu1—Cu2	85.42 (9)	C12—C13—H13B	109.5
N1—Cu1—Cu2	178.11 (9)	H13A—C13—H13B	109.5
O6—Cu2—O4	170.13 (12)	C12—C13—H13C	109.5
O6—Cu2—O8	89.16 (14)	H13A—C13—H13C	109.5
O4—Cu2—O8	89.27 (14)	H13B—C13—H13C	109.5
O6—Cu2—O2	91.47 (14)	C12—C14—H14A	109.5
O4—Cu2—O2	87.93 (14)	C12—C14—H14B	109.5
O8—Cu2—O2	167.25 (13)	H14A—C14—H14B	109.5
O6—Cu2—N2	93.19 (13)	C12—C14—H14C	109.5
O4—Cu2—N2	96.66 (12)	H14A—C14—H14C	109.5
O8—Cu2—N2	95.82 (12)	H14B—C14—H14C	109.5
O2—Cu2—N2	96.86 (13)	C12—C15—H15A	109.5
O6—Cu2—Cu1	83.59 (9)	C12—C15—H15B	109.5
O4—Cu2—Cu1	86.57 (9)	H15A—C15—H15B	109.5
O8—Cu2—Cu1	84.71 (9)	C12—C15—H15C	109.5
O2—Cu2—Cu1	82.71 (9)	H15A—C15—H15C	109.5
N2—Cu2—Cu1	176.73 (9)	H15B—C15—H15C	109.5
C1—O1—Cu1	121.5 (3)	O7—C16—O8	125.4 (4)
C1—O2—Cu2	124.5 (3)	O7—C16—C17B	116.5 (4)
C6—O3—Cu1	125.2 (3)	O8—C16—C17B	118.1 (4)
C6—O4—Cu2	120.2 (3)	O7—C16—C17A	116.5 (4)
C11—O5—Cu1	121.1 (3)	O8—C16—C17A	118.1 (4)
C11—O6—Cu2	124.4 (3)	C18A—C17A—C19A	114.0 (5)
C16—O7—Cu1	124.0 (3)	C18A—C17A—C16	112.3 (4)
C16—O8—Cu2	121.8 (3)	C19A—C17A—C16	113.6 (4)
C25—N1—C21	117.7 (4)	C18A—C17A—C20A	106.2 (5)
C25—N1—Cu1	120.0 (3)	C19A—C17A—C20A	105.8 (6)
C21—N1—Cu1	121.7 (3)	C16—C17A—C20A	103.8 (4)
C26—N2—C30	118.3 (4)	C20B—C17B—C18B	123.4 (12)
C26—N2—Cu2	121.4 (3)	C20B—C17B—C16	111.2 (9)
C30—N2—Cu2	120.3 (3)	C18B—C17B—C16	113.3 (9)
O2—C1—O1	124.9 (4)	C20B—C17B—C19B	104.5 (12)
O2—C1—C2	118.5 (4)	C18B—C17B—C19B	103.3 (12)
O1—C1—C2	116.6 (4)	C16—C17B—C19B	96.6 (8)
C3—C2—C5	111.3 (5)	C17B—C18B—H18D	109.5
C3—C2—C1	107.2 (4)	C17B—C18B—H18E	109.5
C5—C2—C1	111.0 (4)	H18D—C18B—H18E	109.5
C3—C2—C4	109.5 (5)	C17B—C18B—H18F	109.5
C5—C2—C4	109.1 (5)	H18D—C18B—H18F	109.5
C1—C2—C4	108.7 (4)	H18E—C18B—H18F	109.5
C2—C3—H3A	109.5	C17B—C19B—H19D	109.5
C2—C3—H3B	109.5	C17B—C19B—H19E	109.5
H3A—C3—H3B	109.5	H19D—C19B—H19E	109.5
C2—C3—H3C	109.5	C17B—C19B—H19F	109.5
H3A—C3—H3C	109.5	H19D—C19B—H19F	109.5
H3B—C3—H3C	109.5	H19E—C19B—H19F	109.5
C2—C4—H4A	109.5	C17B—C20B—H20D	109.5
C2—C4—H4B	109.5	C17B—C20B—H20E	109.5
H4A—C4—H4B	109.5	H20D—C20B—H20E	109.5
C2—C4—H4C	109.5	C17B—C20B—H20F	109.5
H4A—C4—H4C	109.5	H20D—C20B—H20F	109.5
H4B—C4—H4C	109.5	H20E—C20B—H20F	109.5
C2—C5—H5A	109.5	N1—C21—C22	122.8 (4)
C2—C5—H5B	109.5	N1—C21—H21	118.6
H5A—C5—H5B	109.5	C22—C21—H21	118.6
C2—C5—H5C	109.5	C21—C22—C23	118.9 (4)
H5A—C5—H5C	109.5	C21—C22—H22	120.6
H5B—C5—H5C	109.5	C23—C22—H22	120.6
O3—C6—O4	125.6 (4)	C24—C23—C22	118.9 (4)
O3—C6—C7	117.1 (4)	C24—C23—H23	120.5
O4—C6—C7	117.2 (3)	C22—C23—H23	120.5
C10—C7—C8	110.0 (4)	C23—C24—C25	118.5 (4)
C10—C7—C9	109.8 (4)	C23—C24—H24	120.7
C8—C7—C9	110.2 (4)	C25—C24—H24	120.7
C10—C7—C6	111.2 (4)	N1—C25—C24	123.1 (4)
C8—C7—C6	110.3 (3)	N1—C25—H25	118.4
C9—C7—C6	105.3 (3)	C24—C25—H25	118.4
C7—C8—H8A	109.5	N2—C26—C27	123.2 (4)
C7—C8—H8B	109.5	N2—C26—H26	118.4
H8A—C8—H8B	109.5	C27—C26—H26	118.4
C7—C8—H8C	109.5	C26—C27—C28	118.3 (4)
H8A—C8—H8C	109.5	C26—C27—H27	120.9
H8B—C8—H8C	109.5	C28—C27—H27	120.9
C7—C9—H9A	109.5	C29—C28—C27	119.3 (4)
C7—C9—H9B	109.5	C29—C28—H28	120.4
H9A—C9—H9B	109.5	C27—C28—H28	120.4
C7—C9—H9C	109.5	C28—C29—C30	118.4 (4)
H9A—C9—H9C	109.5	C28—C29—H29	120.8
H9B—C9—H9C	109.5	C30—C29—H29	120.8
C7—C10—H10A	109.5	N2—C30—C29	122.6 (4)
C7—C10—H10B	109.5	N2—C30—H30	118.7
H10A—C10—H10B	109.5	C29—C30—H30	118.7
			
O7—Cu1—Cu2—O6	−89.67 (15)	Cu2—O2—C1—C2	−179.0 (3)
O1—Cu1—Cu2—O6	89.73 (15)	Cu1—O1—C1—O2	−4.5 (6)
O3—Cu1—Cu2—O6	178.42 (15)	Cu1—O1—C1—C2	175.5 (3)
O5—Cu1—Cu2—O6	−0.09 (15)	O2—C1—C2—C3	−109.5 (5)
O7—Cu1—Cu2—O4	89.67 (15)	O1—C1—C2—C3	70.5 (6)
O1—Cu1—Cu2—O4	−90.93 (15)	O2—C1—C2—C5	12.2 (7)
O3—Cu1—Cu2—O4	−2.25 (15)	O1—C1—C2—C5	−167.8 (5)
O5—Cu1—Cu2—O4	179.25 (14)	O2—C1—C2—C4	132.2 (5)
O7—Cu1—Cu2—O8	0.08 (14)	O1—C1—C2—C4	−47.8 (6)
O1—Cu1—Cu2—O8	179.48 (15)	Cu1—O3—C6—O4	3.0 (7)
O3—Cu1—Cu2—O8	−91.83 (15)	Cu1—O3—C6—C7	−172.9 (3)
O5—Cu1—Cu2—O8	89.66 (15)	Cu2—O4—C6—O3	−5.9 (6)
O7—Cu1—Cu2—O2	178.01 (15)	Cu2—O4—C6—C7	170.0 (3)
O1—Cu1—Cu2—O2	−2.58 (15)	O3—C6—C7—C10	−158.3 (4)
O3—Cu1—Cu2—O2	86.10 (15)	O4—C6—C7—C10	25.4 (5)
O5—Cu1—Cu2—O2	−92.41 (15)	O3—C6—C7—C8	−36.1 (5)
O3—Cu1—O1—C1	−77.8 (4)	O4—C6—C7—C8	147.7 (4)
O5—Cu1—O1—C1	89.8 (4)	O3—C6—C7—C9	82.8 (5)
N1—Cu1—O1—C1	−174.3 (4)	O4—C6—C7—C9	−93.5 (4)
Cu2—Cu1—O1—C1	4.4 (3)	Cu1—O5—C11—O6	3.2 (6)
O6—Cu2—O2—C1	−81.5 (4)	Cu1—O5—C11—C12	−174.4 (3)
O4—Cu2—O2—C1	88.6 (4)	Cu2—O6—C11—O5	−3.4 (7)
O8—Cu2—O2—C1	11.2 (9)	Cu2—O6—C11—C12	174.2 (3)
N2—Cu2—O2—C1	−174.9 (4)	O5—C11—C12—C13	−140.4 (4)
Cu1—Cu2—O2—C1	1.8 (3)	O6—C11—C12—C13	41.7 (5)
O7—Cu1—O3—C6	−83.2 (4)	O5—C11—C12—C14	100.5 (5)
O1—Cu1—O3—C6	87.0 (4)	O6—C11—C12—C14	−77.4 (5)
O5—Cu1—O3—C6	7.5 (9)	O5—C11—C12—C15	−18.6 (6)
N1—Cu1—O3—C6	−178.0 (4)	O6—C11—C12—C15	163.6 (4)
Cu2—Cu1—O3—C6	0.6 (4)	Cu1—O7—C16—O8	−0.9 (6)
O8—Cu2—O4—C6	89.4 (3)	Cu1—O7—C16—C17B	177.9 (3)
O2—Cu2—O4—C6	−78.2 (3)	Cu1—O7—C16—C17A	177.9 (3)
N2—Cu2—O4—C6	−174.8 (3)	Cu2—O8—C16—O7	1.0 (6)
Cu1—Cu2—O4—C6	4.7 (3)	Cu2—O8—C16—C17B	−177.8 (3)
O7—Cu1—O5—C11	82.7 (4)	Cu2—O8—C16—C17A	−177.8 (3)
O1—Cu1—O5—C11	−87.5 (4)	O7—C16—C17A—C18A	39.7 (6)
O3—Cu1—O5—C11	−8.2 (9)	O8—C16—C17A—C18A	−141.4 (5)
N1—Cu1—O5—C11	177.2 (4)	C17B—C16—C17A—C18A	0(100)
Cu2—Cu1—O5—C11	−1.4 (3)	O7—C16—C17A—C19A	171.0 (5)
O8—Cu2—O6—C11	−83.1 (4)	O8—C16—C17A—C19A	−10.1 (7)
O2—Cu2—O6—C11	84.2 (4)	C17B—C16—C17A—C19A	0(82)
N2—Cu2—O6—C11	−178.9 (4)	O7—C16—C17A—C20A	−74.6 (6)
Cu1—Cu2—O6—C11	1.7 (3)	O8—C16—C17A—C20A	104.2 (5)
O3—Cu1—O7—C16	82.3 (4)	C17B—C16—C17A—C20A	0(100)
O5—Cu1—O7—C16	−85.1 (4)	O7—C16—C17B—C20B	−150.8 (10)
N1—Cu1—O7—C16	179.0 (3)	O8—C16—C17B—C20B	28.1 (11)
Cu2—Cu1—O7—C16	0.3 (3)	C17A—C16—C17B—C20B	0(4)
O6—Cu2—O8—C16	83.1 (3)	O7—C16—C17B—C18B	−6.7 (11)
O4—Cu2—O8—C16	−87.2 (3)	O8—C16—C17B—C18B	172.2 (11)
O2—Cu2—O8—C16	−9.9 (9)	C17A—C16—C17B—C18B	0(100)
N2—Cu2—O8—C16	176.2 (3)	O7—C16—C17B—C19B	100.8 (8)
Cu1—Cu2—O8—C16	−0.5 (3)	O8—C16—C17B—C19B	−80.3 (9)
O7—Cu1—N1—C25	52.8 (3)	C17A—C16—C17B—C19B	0(100)
O1—Cu1—N1—C25	−126.9 (3)	C25—N1—C21—C22	1.2 (7)
O3—Cu1—N1—C25	144.4 (3)	Cu1—N1—C21—C22	−169.9 (4)
O5—Cu1—N1—C25	−36.8 (4)	N1—C21—C22—C23	−0.5 (8)
O7—Cu1—N1—C21	−136.4 (4)	C21—C22—C23—C24	0.1 (7)
O1—Cu1—N1—C21	44.0 (4)	C22—C23—C24—C25	−0.4 (7)
O3—Cu1—N1—C21	−44.8 (4)	C21—N1—C25—C24	−1.6 (7)
O5—Cu1—N1—C21	134.0 (3)	Cu1—N1—C25—C24	169.6 (4)
O6—Cu2—N2—C26	32.0 (3)	C23—C24—C25—N1	1.2 (7)
O4—Cu2—N2—C26	−147.4 (3)	C30—N2—C26—C27	1.2 (6)
O8—Cu2—N2—C26	−57.5 (3)	Cu2—N2—C26—C27	178.6 (3)
O2—Cu2—N2—C26	123.9 (3)	N2—C26—C27—C28	−2.1 (7)
O6—Cu2—N2—C30	−150.6 (3)	C26—C27—C28—C29	1.6 (7)
O4—Cu2—N2—C30	29.9 (3)	C27—C28—C29—C30	−0.4 (7)
O8—Cu2—N2—C30	119.9 (3)	C26—N2—C30—C29	0.1 (6)
O2—Cu2—N2—C30	−58.8 (3)	Cu2—N2—C30—C29	−177.4 (3)
Cu2—O2—C1—O1	1.0 (7)	C28—C29—C30—N2	−0.5 (7)

### Hydrogen-bond geometry (Å, °)

**Table:**

Cg1 and Cg2 are the centroids of the N1,C21–C25 and N2,C26–C30 rings, respectively.

**Table:**

*D*—H···*A*	*D*—H	H···*A*	*D*···*A*	*D*—H···*A*
C3—H3c···Cg1^i^	0.98	2.90	3.609 (7)	130
C19b—H19f···Cg2^ii^	0.98	2.64	3.554 (19)	154

Symmetry codes: (i) *x*−1, *y*, *z*; (ii) *x*+1, *y*, *z*.
